# Comparison of pre-bent titanium mesh versus polyethylene implants in patient specific orbital reconstructions

**DOI:** 10.1186/1746-160X-9-32

**Published:** 2013-10-29

**Authors:** Marcin Kozakiewicz, Piotr Szymor

**Affiliations:** 1 Department of Maxillofacial Surgery, Medical University of Lodz, ul. Zeromskiego 113, 90-549 Lodz, Poland

**Keywords:** Facial skeleton injuries, Individual implants, Titanium mesh, Ultrahigh molecular weight polyethylene, Diplopia

## Abstract

**Introduction:**

Computerized tomography DICOM file can be relatively easily transformed to a virtual 3D model. With the help of additional software we are able to create the mirrored model of an undamaged orbit and on this basis produce an individual implant for the patient Authors decided to apply implants with any thickness, which are authors own invention to obtain volumetric support and more stable orbital wall reconstruction outcome. Material of choice was ultra-high molecular weight polyethylene (UHMWPE).

**Objective:**

The aim of this study was to present and compare functional results of individual reconstructions of orbital wall using either titanium mesh or ultra-high molecular weight polyethylene.

**Materials and methods:**

57 consecutive patients affected by orbital wall fracture (46 males, 11 females, mean age 34±14 year) were treated in Department of Maxillofacial Surgery from 2010 to 2012. In the first group we used patient specific treatment by titanium mesh shaped on a 3D printed model of a mirrored intact orbit (37 orbits) or by individually manufactured UHMW-PE implantby CAM milling in second group (20 orbits). All of these patients were subjected to preoperative helical computerized tomography and consultation of an ophthalmologist (including binocular single vision loss test - BSVL). Further on, patients were operated under general anaesthesia using transconjuctival approach. BSVL was again evaluated post-operationally in 1 month and 6 months later.

**Results:**

Functional treatment results (BSVL) for both groups were similar in 1 month as well as 6 months post operational time. There was no statistically significant difference between these two groups.

**Conclusions:**

This study of 6 months functional result assessment of pre-bent individual implants and CNC milled ultra-high molecular weight polyethylene of the orbital wall has shown it to be a predictable reconstruction method. Individually shaped UHMWPE seems to be as good as pre-bent titanium mesh.

## Introduction

Orbital wall fractures are quite common consequence of maxillofacial trauma. 3 [1]-32% [2] of all maxillofacial fractures are present within the orbit. Although aetiology of facial trauma varies in different countries the most common causes worldwide are assault, traffic accident, sports and fall. Common complications of such fractures are diplopia and enophtalmos [3,4].

In 2000s individual reconstructions in maxillofacial surgery begun to be more popular [5-12]. Technical equipment and software is more available nowadays. Computerized tomography DICOM file can be relatively easily transformed to a virtual model [8,13]. With the help of additional software the mirrored model of an undamaged orbit can be created and exported as a .stl file for external 3D printer. On such printed model it is possible to bend titanium mesh preoperatively. This allows to reduce operating time, improve safety [13,14] and achieve much better accuracy of orbital reconstruction [9,10,15]. However, sometimes it is observed that implants may become misplaced or deformed, especially in cases of severe damaged orbits and old cases where thin titanium mesh cannot be efficiently supported. Therefore authors decided to use implants of authors own invention with any desired thickness to obtain volumetric support and more stable orbital wall reconstruction outcome [16]. Material of choice was ultra-high molecular weight polyethylene (UHMW-PE) commonly used in medicine [17-19] especially as acetabulum replacement in total hip prosthesis. An initial interest in UHMWE-PE to use it in maxillofacial surgery is dated at 1999 [20]. The problem of shrinkage of the polymer during its processing led to rejection of this material from maxillofacial reconstructions at that time. To eliminate this problem computerized numerical control milling from earlier prepared solid bar of UHMW-PE was suggested. This led to the first successful human application of solid patient specific orbital wall implant made from UHMWE-PE at 2012 [16].

## Objective

The objective of this study was to present and compare functional results of individual reconstructions of orbital wall using either titanium mesh or ultra-high molecular weight polyethylene.

## Material and methods

The Medical University of Lodz Ethic Committee approval was obtained for this study [RNN/266/11/KB, RNN/141/12/KB, RNN/267/11/KB]. Participants provided their written informed consent in a form accepted by Medical University of Lodz Ethic Committee to participate in this study. 57 consecutive patients affected by orbital wall fracture (46 males, 11 females, mean age 34±14 year) were treated in Department of Maxillofacial Surgery from 2010 to 2012. Inclusion criteria was unilateral side injury i.e. 57 reconstructions were performed. In the first group pre-bent titanium mesh (37 orbits) was used and in the second group computerized numerical control milled ultrahigh molecular weight polyethylene [16] implants (20 orbits) were used. Patients to both groups were assigned randomly.

**Figure 1:**
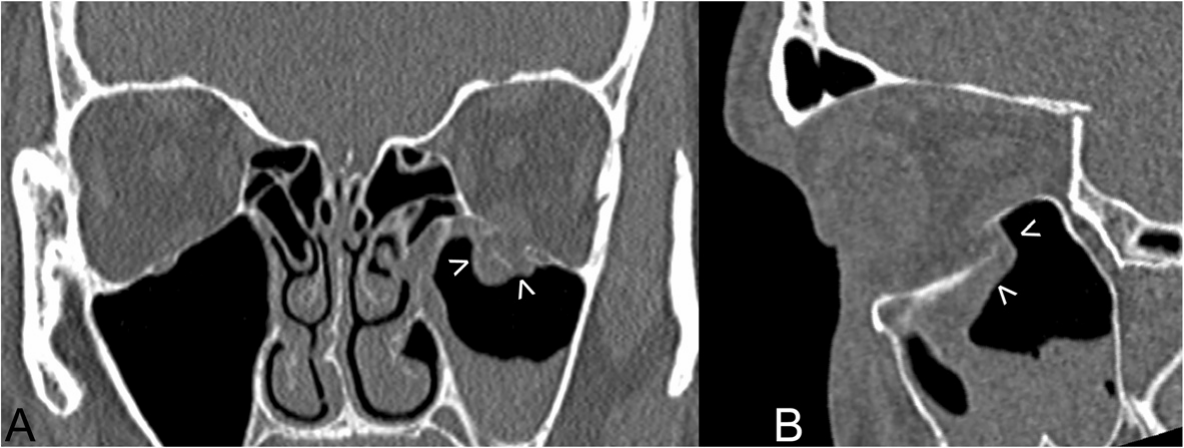
**Blow-out fracture of the left orbit floor. A** - coronal view in computerized tomography. **B** - sagittal view through left orbit. Bone fragments displacement and herniated orbital tissues shown by arrows.

39 of participating patients were diagnosed with fracture of at least one of the orbital walls without any damage to surrounding facial skeleton – isolated orbital wall fracture(IOWF) (Figure 1). 15 of participating patients were diagnosed with zygomatic complex fractures, 3 of them were diagnosed with low energy fracture of zygomatic complex and fracture of one orbital wall - zygomatico-orbital fracture (ZOF) and 12 of them were diagnosed with high energy zygomatic complex fracture involving also fractures of maxillary sinus walls and usually more than one orbital wall - zygomatico-maxillo-orbital fractures (ZMOF). 3 of participating patients suffering from panfacial fractures involving only one orbit were diagnosed as comminuted one-side fractures (COSF) (Table 1). The system of classification of maxillofacial fractures was based on classification by H. Wanyura [21,22].

**Table 1:** Specific to implant type patient data

	**UHMW-PE**	**Titanium mesh**	**Total value**
Total	20	37	57
Males	14	32	46
Females	6	5	11
Mean age	31.35	35.00	
Mean ODI	3.75	2.27	
**Trauma cause**			
Assault	12	19	31
Traffic accident	6	13	19
Fall	2	5	7
**Diagnosis**			
ZMOF	6	6	12
IOMF	12	27	39
ZOF	2	1	3
COSF	0	3	3
Mean BSVL_PRE	27.08%	20.22%	n.s.
Mean BSVL_01	29.20%	20.32%	n.s
Mean BSVL_06	15.95%	15.49%	n.s

*Abbreviations*:

*n.s.* no significant statistical difference.

*UHMW-PE* ultra high molecular weight polyethylene.

*ODI* orbital destruction intensity scale.

*ZMOF* zygomatico-maxillo-orbital fractures.

*IOMF* isolated orbital wall fracture.

*ZOF* zygomatico-orbital bone fracture.

*COSF* comminuted one-side fracture of the orbit.

*BSVL_PRE* preoperative binocular single vision loss test [0% intact vision, 100% double in whole field of view].

*BSVL_01* 1 month postoperative binocular single vision loss test.

*BSVL_06* 6 months postoperative binocular single vision loss test.

The most common cause of the trauma was assault (31 cases), followed by traffic accident (19 cases) and fall (7 cases) (Table 1).

All patients were subjected to preoperative helical computerized tomography by Multi-slice VCT, GE Lightspeed 64-slice scanner using 0.6 mm slice thickness, a gantry tilt of 0° and with a matrix of 512 × 512 pixels, 120 kVp, 115 mAs. The types of injury were then classified by an orbital destruction intensity (ODI) scale [15] to compare the distribution of injury intensity in both groups (Table 1).

**Figure 2:**
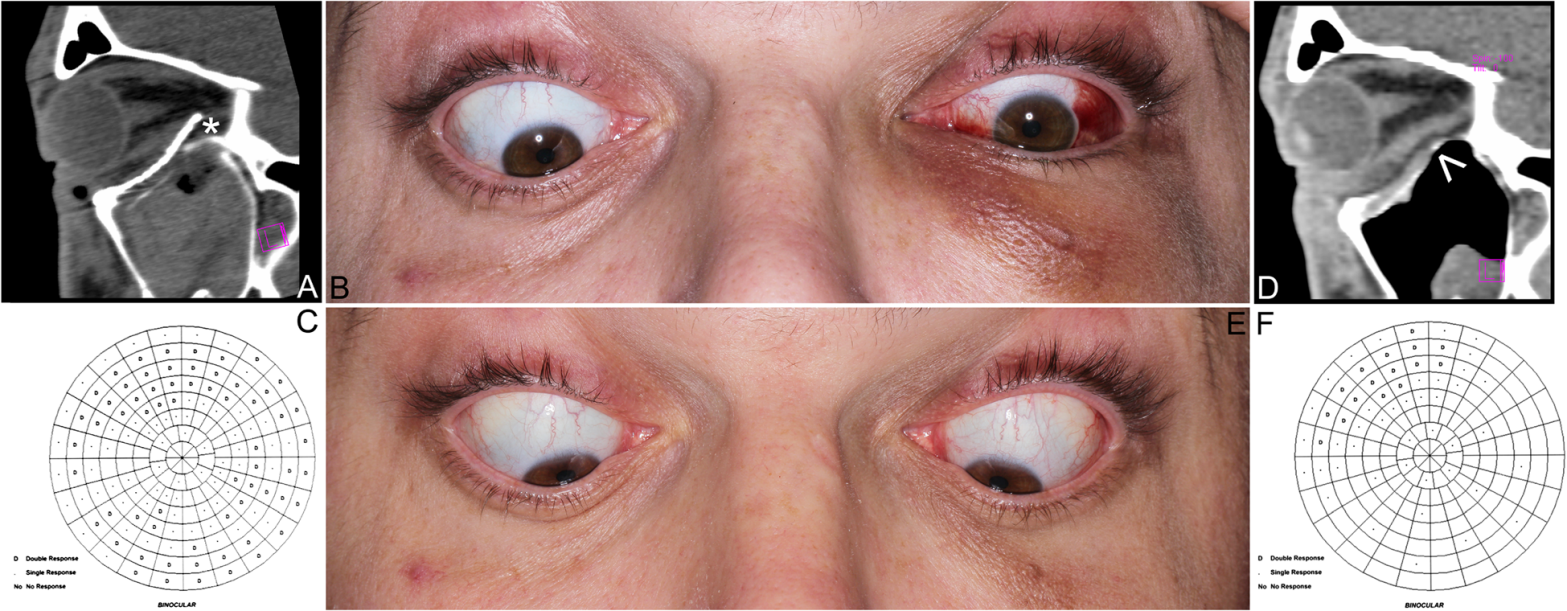
**Results of treatment. A** – computerized tomography in sagittal view: titanium mesh deformated during intra-orbital maneuvres in primary surgery (asterisk); its deepest located part hurts the inferior rectus muscle. **B** – downgaze significantly limited in the left eye; **C** – result of binocular single vision loss test: diplopia caused by titanium mesh is mainly up- and downgaze. **D** - computerized tomography in sagittal view after corrective surgery and exchange of the implant to stiff patient specific ultra-high molecular weight polyethylene implant: orbital floor is reconstructed (arrow) and inferior rectus muscle is free. **E** – normal eye globe motility: full downgaze 1 month post-operationally. **F** - result of binocular single vision loss test: residual diplopia in upgaze/left.

After clinical diagnosis, (Figure 2A, B) a consultation by an ophthalmologist (including binocular single vision test) (Figure 2C) and in case of need a neurosurgeon, patients were qualified to patient specific treatment either by titanium mesh (Synthes, Zuchwil, Switzerland) shaped on a 3D printed model of a mirrored intact orbit [7,15] or by individually manufactured UHMW-PE implant.

Binocular single vision investigation (Figure 2C) was performed using Medmont M600W Automated Perimeter (Medmont Pty Ltd., Nunawading Victoria, Australia) [15]. The patient was asked to decide whether a stimulus seen on perimeter globe appears as a single or a double spot. Pattern of 21 cells was tested, extending 30° superiorly and 40° inferiorly and the result was shown as a percentage of a vision field affected by diplopia (BSVL).

UHMW-PE implants were designed and produced in accordance with the method described previously by the authors [16]. The chosen substrate material was medical UHMW-PE for surgical implants produced in accordance with ISO 5834–1 2007 type 1, ISO 5834–2 2006 type 1 and ASTM F 648–07 type 1 standards (Ticona Engineering Polymers, Florence, USA; http://www.ticona.com). After compression moulding and ram extrusion, material was formed into stock shapes or solid blocks, as necessary for milling. Designing implants began with segmenting acquired DICOM data using Amira 5.4 (Visage Imaging GmbH, Germany) and creating 3D model of the patient’s facial skeleton. In the next stage with use of Geomagic Studio 11 (Geomagic Corp., Morrisville, USA), a mirrored model of the unaffected side was superimposed on model of fractured side. To ensure proper alignment analysis of symmetry was performed. The reference areas were undamaged upper rim and upper wall. Proper superimposition allowed creating superior (from undamaged mirrored orbit) and inferior (from model of fractured orbit) surfaces of implant. Subsequently, the 3D model was transferred to a CAD program SolidWorks (Dassault Systèmes SolidWorks Corp., Waltham, USA) and prepared for CNC milling. Each virtual implant was inspected and approved after necessary corrections by a maxillofacial surgeon (MK) before manufacturing. All UHMW-PE implants were produced on computer numerical controlled, 5-axis milling machine Speed Hawk 650 (OPS-Ingersoll Funkenerosion GmbH, Burbach, Germany) with accuracy of 0.05 mm.

**Figure 3:**
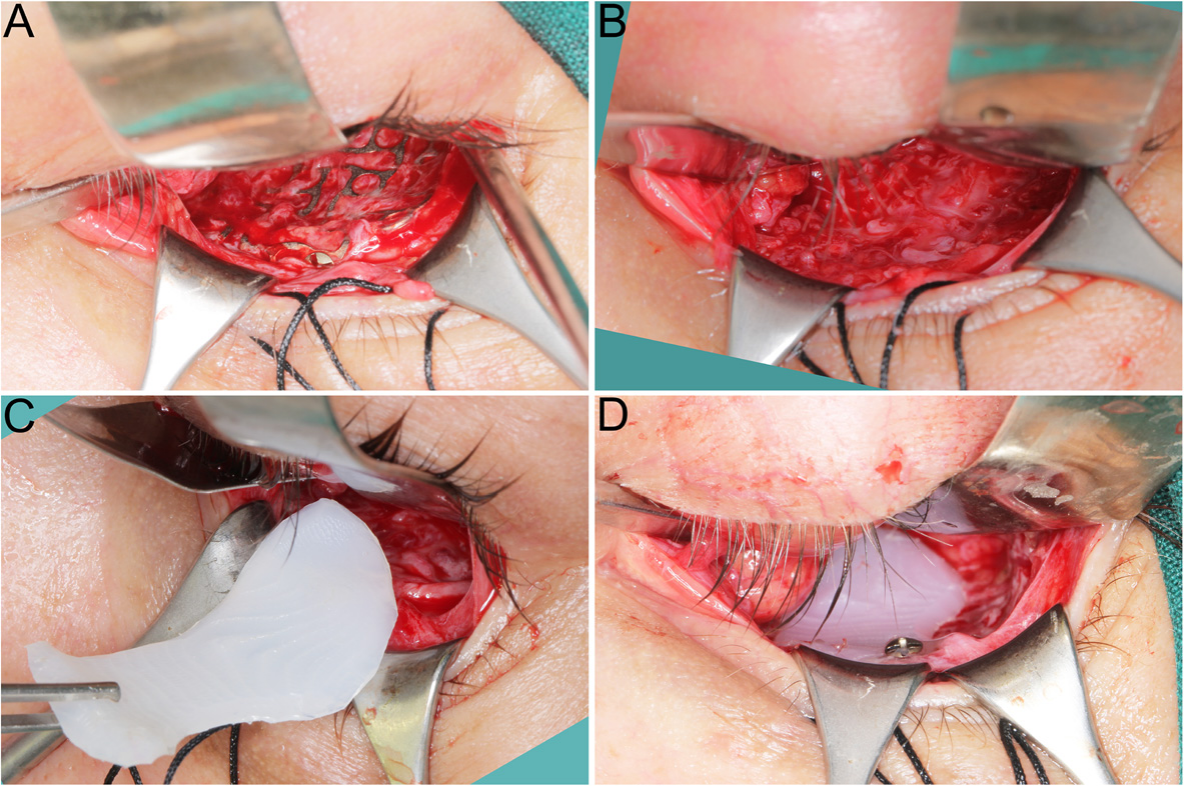
**Surgical steps in exchange titanium mesh to individual ultra-high molecular weight polyethylene implant. A** – transconjunctival approach exposes the titanium mesh immerse in scar tissue in orbital floor. Orbital spatula holding the globe in the upper section, hooks holding the eylid in the lower part of the picture. **B** – mesh impressions in orbital floor scar tissue. **C** – individual polyethylene implant insertion through the transconjunctival approach. **D** – implant position in the orbit, implant alignment checked in previously designed reference areas, here visible fit of the implant to lower orbital rim. Implant position fixed with single 6mm long self-tapping screw from MatrixMIDFACE system by SYNTHES (Synthes, Zuchwil, Switzerland).

Further on, patients were operated under general anaesthesia by the same surgeon (author MK) (Figure 3). Transconjunctival approach was used in all cases (Figure 3A). In the first group flat titanium 0.4 mm thick mesh was shaped preoperatively by operating surgeon(MK) on a solid individual model [Ti-Mesh] [7,15]. In the next group previously prepared ultrahigh molecular weight polyethylene [UHMW-PE] [16] implants were used to reconstruct affected lower or lower and medial wall of the orbit. The correctness of position of implant during operation was controlled by checking implant alignment in previously designed reference areas (usually lower orbital rim anteriorly and orbital process of palatal bone posteriorly). In both groups the anatomical orbital wall reconstruction were obtained. Computerized tomography was performed in the first week after surgery (Figure 2D) to evaluate the quality of the reconstruction and the condition of surrounding tissues to exclude any complications. Binocular single vision was again evaluated post-operationally in 1 month and 6 months later (Figure 2F).

Collected data were statistically analysed in Statgraphics Centurion XVI (STATPOINT TECHNOLOGIES, INC., Warrenton, Virginia, USA) (summary statistics, ANOVA, analysis of linear regression, t-test). Statistical significance was determined as p< 0.05.

## Results

**Figure 4:**
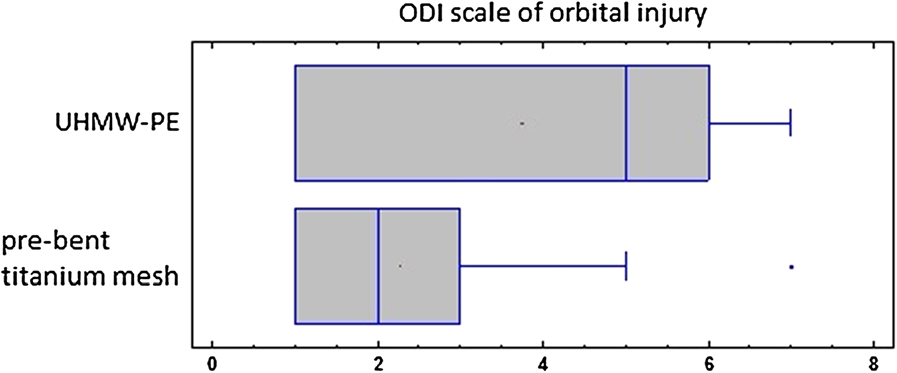
**Plot comparison of ODI scale [**[15]**] of orbital injury and material used for orbital walls reconstruction.** Although patients were assigned to both groups randomly there was a statistically significant difference between those two groups. Patients treated with ultra-high molecular weight polyethylene suffered from more extensive fractures than those treated with pre-bent titanium mesh. ODI scale [15] is described as follows: 1. site of destruction: floor i.e. one wall (1W); 2. floor+one wall (medial or lateral) i.e. two walls (2W); 3. floor+one margin i.e. one wall and one orbital margin (1W+1M);4. floor+one wall+one margin i.e. 2W+1M; 5. floor+one wall+two margins i.e. 2W+2M; 6. floor+two walls+one margin i.e. 3W+1M; 7. floor+one or two walls+two margins i.e. 3W+2M; 8. floor+two or three walls+more than one margin i.e. 3-4W+2-4M.

Although patients were assigned to both groups at random there was a statistically significant difference between mean ODI value in polyethylene and titanium mesh group (p<0.01). There was a higher orbital wall destruction in a group treated with UHMW-PE (Figure 4). Despite that both groups were similar as far as pre-treatment BSVL is considered. There was a statically significant difference between mean ODI value between fractures of left (ODI=2) and right (ODI=4) side of the face (ANOVA p<0.00001).

**Figure 5:**
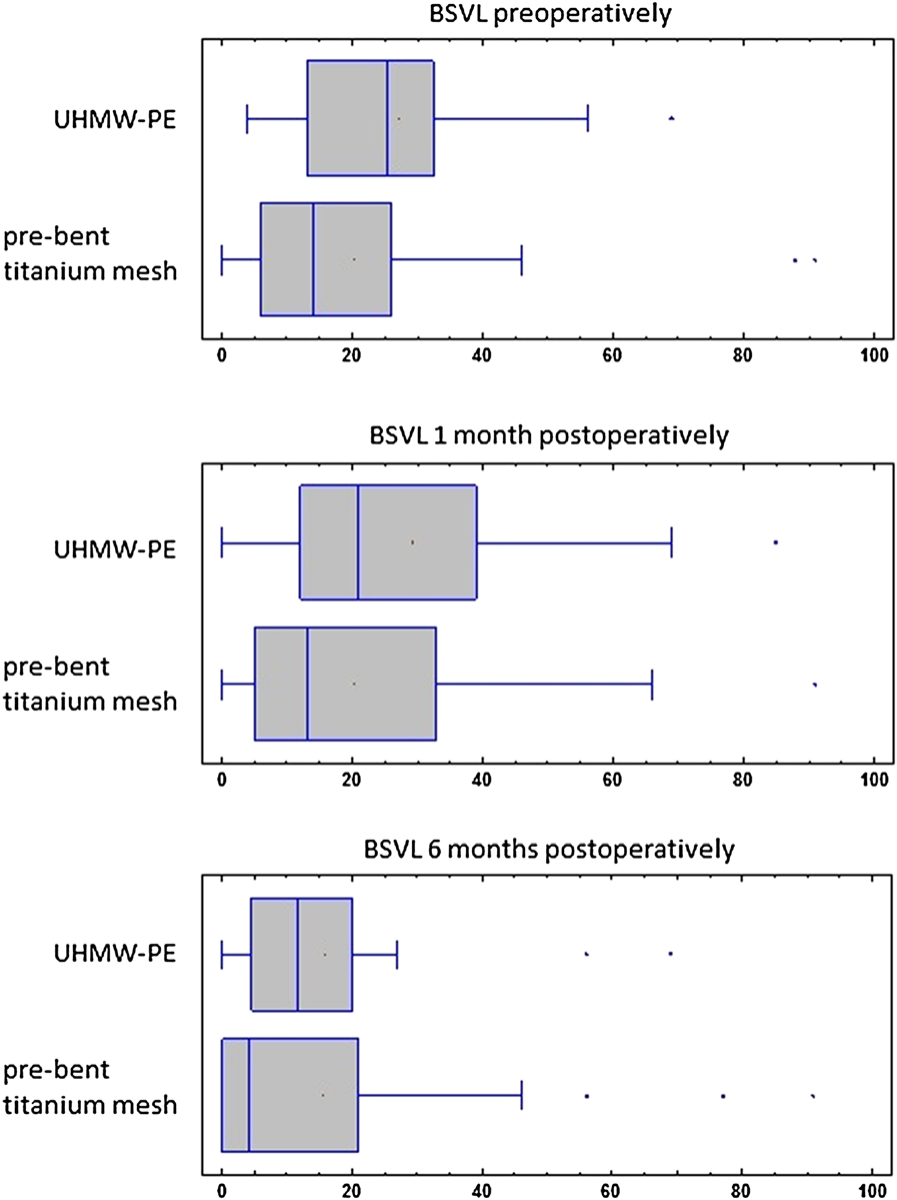
**Comparison of treatment results in percentage of field of vision affected with loss of single vision (BSVL) for ultra-high molecular weight polyethylene and pre-bent titanium mesh.** Examination was performed preoperatively, 1 month postoperatively and 6 months postoperatively. Despite differences in scale of injury in both groups (Figure 4) there was no statistically important difference in treatment results between patients treated with pre-bent titanium mesh or individually shaped CNC milled UHMW-PE implants.

Despite the differences treatment results (BSVL) for both groups were similar in 1 month as well as 6 months post operational time. For pre-bent titanium mesh mean BSVL preoperational was 20.22%, 1 month after operation it was 20.32% and 6 months after the operation lowered to 15.48%. For UHMW-PE these values were 27.07% preoperational, 29.2% 1 month post operational and 15.95% 6 months post operational. There was no statistically significant difference between these two groups (Figure 5). There was no statistically significant difference between groups divided by the cause of trauma neither in preoperative nor in late postoperative results.

**Figure 6:**
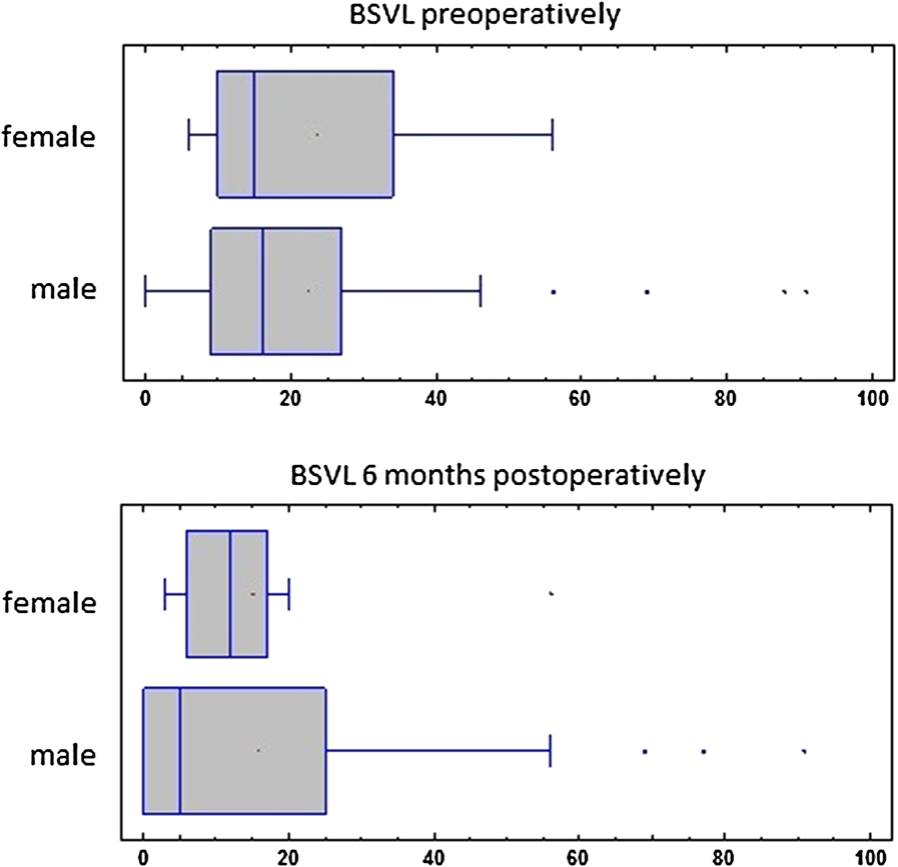
**Plot comparison of percentage of field of vision affected with loss of single vision (BSVL) pre- and 6 months postoperatively in accordance to patients’ gender.** There was no statistically important difference in treatment results between genders.

In this study 80.7% of patients with orbital wall fracture were male what coincides with results of other studies [3,23,24]. Gender had no statistically significant influence on BSVL preoperatively or on treatment results (Figure 6).

Clinical classification of a fracture has a statistically significant impact on pre-operational BSVL results. Patients who suffered from isolated orbital wall fracture, or zygomato-orbital bone fracture, had a mean BSVL much lower (18.09%; 12.83%) than those who suffered from zygomatico-maxillo-orbital fractures or comminuted one-side fractures (36.96%; 34.0%). These statistically important differences between groups disappear in early and late post operational results, where there is no statistically significant difference in treatment results depending on primary clinical diagnosis.

There is a statistically significant relationship between ODI scale of injury and preoperative BSVL (p<0.001, correlation coefficient = 0.47), early postoperative BSVL (p<0.005, correlation coefficient = -0.42) or late postoperative BSVL (p<0.005, correlation coefficient = 0.42). It is not surprising that with the rise of the ODI scale of injury there is also a rise in diplopia value [BSVL] not only preoperatively but also postoperatively.

Although there is no correlation between patients’ age and BSVL preoperatively, there is a relatively weak but significant relationship between age and early postoperative BSVL (p<0.05, correlation coefficient = -0.29) or late postoperative BSVL (p=0.05, correlation coefficient = -0.27). The older the patient was, the decrease of BSVL postoperatively was poorer.

## Discussion

Within the last few years CAD and CAM in reconstruction of orbital fractures has become a commonly used technique [7,15,25,26]. New materials [27-31], new methods of constructing patient-specific implants [8,9,25,32-34] of fractured orbital walls are introduced each year. Each of these innovations has the aim to produce quicker, cheaper and better fitted implants. Most common materials used for orbital wall reconstruction worldwide are autologous bone grafts [30,34-36], porous polyethylene [37-41], and polydioxanone (PDS) [28,30,42]. Titanium mesh has already proven its usefulness in reconstructing orbital walls [35,43]. Alternative materials for orbital walls reconstruction such as hydroxyapatite, porous polyethylene [37] or polylactide provide as good treatment results as titanium mesh [30]. Ultra high molecular weight polyethylene used in this study has proven to be as useful in reconstructing orbital walls as pre-bent titanium mesh. No statistical differences in post operational results depending gender, age or primary clinical diagnosis show that polymers may be broadly used instead of titanium mesh. An ability to create implants with nearly any thickness due to CNC milling seems to be helpful in reconstructing heavily destroyed orbits and especially in delayed surgery cases. It is possible to adjust implant thickness to fully recreate orbital walls. Costs of producing ultra-high molecular weight polyethylene implants are similar to using titanium mesh, decreased of a cost of 3D printed acrylic model of an orbit. Compared to porous polyethylene [30,41] UHMW-PE implants should probably have lower a risk of infection due to their solid structure. Therefore an elevated risk of deep implant infection before completing vascularization should not occur as there is no vascular ingrowth into implant. In our study there was no case of postoperational implant infection but further studies concerning this issue are required.

A major drawback of polyethylene implants is their radiolucency. It is required to use radio-opaque agent combined with the polyethylene to make implants visible on computed tomography for post-operational control of implant position.

## Conclusions

This study of 6 months functional result assessment of pre-bent individual implants and CNC milled ultra-high molecular weight polyethylene of the orbital wall has shown it to be a predictable reconstruction method. UHMW-PE implant seems to be as good as pre-bent titanium mesh.

## Competing interest

The authors declare that they have no competing interests.

## Authors’ contributions

MK designed the study, preformed all of the operations and statistically analysed the results, also helped to draft the manuscript. PS made literature search, gathered the results and drafted the manuscript. Both authors read and approved the final manuscript.
